# Selections

**Published:** 1816-11

**Authors:** 


### PART III.

### SELECTIONS.

—— Factorum est copia nobis.

### FRENCH DICTIONARY OF MEDICAL SCIENCES.

( Continued from page 347. )

*Medical Jurisprudence.*—Article, *Avortement*;* Lat. *Abortus*; Engl. *Abortion*; Germ. *Unzeitige Niederkunst*—*Misgebarung*; It. *Abortamento*. We have here to consider the subject under its relations with Medical Jurisprudence.

*Sed jacet aurato vix ulla puerpera lecto,*

*Tantùm artes hujus, tantum medicamina possunt,*

*Quæ steriles facit, atque homines in ventre necandos*

*Conducit.*

JUVENAL.

*Vestra quid effoditis subjectis viscera telis,*

*Et nondum natis dura venena datis?*

*Quæ prima instituit teneros convellere foetus*

*Malitiâ fuerat digna perire suâ.*

*Hoc neque in Armeniis tigres fecère latebris,*

*Perdere nec foetus ausa leæna suos.*

*At teneræ faciunt, sed non impunè, puellæ,*

*Sæpè suos quæ utero necat, ipsa perit.*

OVID.

*Definition of the crime.* The employment of any means intended to destroy the fœtus *in utero*, or to procure its expulsion previously to the period at which it is capable of existing, independent of its connections with the mother, constitutes the crime of provoked abortion. This definition obviously excludes the abortion consequent on the employment of medicine, or infliction of violence, taken or incurred without criminal design against the offspring.

Abortion may be involuntary, as induced by the operation of natural causes, or premeditated and determined by external violence. It is the province of the juridical physician to decide whe-

---

* On this, as on all other occasions, we shall not confine ourselves to the French Dictionary; but glean from all the other reputable works on Medical Jurisprudence to which we have access. EDIT.

ther, in any suspected case, the event has taken place accidentally, or been the consequence of criminal proceeding; whether, and by what means, the fœtus has been prematurely expelled; and, lastly, whether, at the period of employment of such means, the fœtus were endued with life.

But previously to our entering upon an exposition of the general principles by which the solution of these important queries may be directed, it may be right to consider the causes by which abortion, spontaneous or voluntary, is commonly produced, and the external and internal signs from which its occurrence may be very confidently inferred.

Spontaneous or involuntary abortion may arise from causes operating directly either on the mother, or the fœtus and its appendages. Under the first class of causes, may be enumerated peculiarity of constitution; moral affections, particularly grief* and the other depressing passions, surprize, terror, sympathy, sometimes excited by the view of another woman in labour; agents which exert a physical operation, as undue plethora, weakness, and irritability; inordinate exertion of any kind, excessive evacuations, intemperance, acute and violent diseases, especially those of the uterus or organs composing the generative and urinary systems, or mechanical injury. Maladies of the fœtus or a morbid condition of the funis, the membranes, or placenta, belong to the second class.

The means by which artificial or voluntary abortion may be determined are either constitutional, as exciting a general disturbance in the economy; or local, and exerting a mechanical operation. The first are by far the least certain in their effects. They consist principally of blood-letting, evacuations from the mouth and rectum, diuretics and emmenagogues. We shall cursorily consider these different agents, and the rank which they respectively hold in the catalogue of abortives.

*Blood-letting. Mulier, in utero ferens, sectâ venâ abortit*, is one of the aphorisms of the venerable Hippocrates: and a similar opinion generally prevails among the people. But abstraction of blood, although when largely employed in a weak, delicate, and irritable female, or otherwise injudiciously resorted to, it may operate in provoking abortion; under other circumstances, and especially in plethoric subjects, is often successfully prescribed to obviate the impending danger. Copious bleeding from the *foot* in the earlier stages of pregnancy is commonly considered more likely to induce disastrous consequences than the same operation performed in the arm, and at a more advanced period of utero-gestation.

*Emetics and Purgatives.* These act either by the general commotion which they excite in the system, or the irritation produced

* This will be particularly liable to operate on an unfortunate female about to give birth to an illegitimate child, and whose mind is keenly alive to the shame and wretchedness of her situation. Edit.

by them in the intestinal canal, and hence propagated to the hypogastric organs. Yet a vomit or aperient, seasonably administered, may remove intestinal disorder, which, otherwise, might have eventually determined abortion. When gastric or alvine evacuants are administered with this view, those of the mildest operation should be selected, and great precaution exercised in their employment.* They, on the contrary, who use such remedies with criminal intention, most commonly choose such as are distinguished by the violence of their action; and take, or administer them in immoderate doses, independently of all necessity or indication.

*Diuretics and Emmenagogues.* Various medicinal substances, which act directly on the urinary organs, are regarded as capable of provoking the menstrual flux, and consequently abortion. But it may be doubted, whether any diuretic or emmenagogue, with the exception of the *lytta*, really possesses any such baneful property. Preparations of savin, and the terebinthinates, have often been very largely administered without inducing these consequences. Electricity, when applied in considerable force, may, particularly in subjects otherwise predisposed to abortion, excite a disturbance in the uterine system, destructive to the healthy performance of its gestatory functions. This will belong to the class of constitutional or local means, according to the mode of its application.

Infliction of blows upon the abdominal or lumbar regions, or other external violence, and the introduction of sharp instruments from the vagina into the uterus, whereby the membranes of the ovum are ruptured, constitute the local and mechanical means usually employed to procure abortion. The former acts by inducing detachment of the placenta, hæmorrhage from the uterine vessels, and the death of the fœtus. But the latter mean is the most certain in its effects, and, repugnant as it may seem to every feeling of the human heart, most commonly employed. As this dreadful crime, however, is usually perpetrated by an ignorant and inexpert operator, the body of the fœtus, or generative organs of the mother, must often exhibit such marks of violence as will lead to its detection, and the punishment of the wretch by whom not only the child, but sometimes its ill-fated mother also, has been remorselessly sacrificed.†

The *signs*, denoting that abortion has taken place, are external and internal; respectively appreciable by common examination and anatomical inspection.—*External.* These may be distinguished as occurring either anteriorly, or subsequently, to the period of abor-

---

* We have seen premature delivery brought on by a very moderate dose of calomel and rhubarb: and abortion, by employment of diuretics, particularly the *lytta*. Hence medical men should be very cautious in administering these substances to pregnant females. Edit.

† See *The remarkable Trial of William Pizzy and Mary Codd*, &c. 8vo. Ipswich, 1808: or a very able Review of it in the sixth volume of the *Edinburgh Medical and Surgical Journal.* Edit.

tion. The anterior signs are cessation of the menstrual flux, depraved appetite, vomiting, protuberant abdomen, and all the other phænomena which denote pregnancy : the subsequent, commonly, are the following: Issue of a watery or bloody kind of milk from the mammæ (when the woman survives the process); sudden subsidence and diminution of these glands; areolæ of the nipples, unusually large and dark: discharge of an ichorous blood from the vagina, sometimes mixed with coagula and mucus; labia soft, red, and swollen; vagina excessively dilated; os uteri gaping, flattened, and inclining downwards; abdominal integuments wrinkled and flaccid; sometimes a disagreeable smell. The woman, moreover, feels pains about the uterine region, shivering and tremors in the extremities, and lassitude. She evinces a frequent wish to lie down, and totters in her walk. The legs sometimes swell; the superficial veins disappear; and the externel parts are discoloured. In addition, it should be remembered, that the hæmorrhage which succeeds abortion or delivery, is more profuse and of longer duration than the menstrual discharge; and that, unlike the latter in its healthy state, the hæmorrhage produces great languor and exhaustion, and forms coagula rarely or never seen in the menstrual blood. These signs, when presenting themselves in combination, assume a very decisive character; but, isolately observed, their evidence must be little relied on, as most of them may be consequent upon the various other diseases to which the human female is subject.—*Internal signs.* These, when the death of the mother admits of the necessary examination, are unusual capacity of the uterus, and thickness of its parietes; traces of adhesion of the placenta to the uneven internal surface of the organ; relaxation of the cervix; dilatation of the vagina; considerable diminution of the ligamenta lata; and an appearance, in the ovaries, of the cicatrix of a *corpus luteum.* But it has been contended by some gentlemen, whose arguments are perhaps more ingenious than solid, that, in any suspected case, "the distension of the uterus might arise from hydatids, or moles, and the inequality of its internal surface (be) occasioned by their attachment."* And, again, it is asserted by practitioners of high authority, that traces of *corpora lutea* sometimes exist in virgins, and in salacious women who have never been impregnated. All these phænomena, it should be recollected, ex-

---

* See "*The Trial of Charles Angus, Esq. for the Murder of Margaret Burns.*" 8vo. Liverpool, 1808; and the two pamphlets therewith connected. A very full account of them is given in the fifth volume of the Edinburgh Med. and Surg. Journal. The writer of that article very judiciously remarks, that, "in cases of doubtful parturition, the proofs are rather to be expected in the appendages of the uterus, in the ovaries, Fallopian tubes, vagina, and areola of the nipples, than in the uterus itself. The appearances in the uterus may be the effect of disease; but such disease never produces those changes in other parts, which are the constant concomitants of pregnancy." See also *Dr. Male's Epitome of Forensic Medicine.* page 120.

ternal and internal, will be observed, whether the abortion or premature delivery have been spontaneous, or violently and voluntarily provoked; and will be more strongly marked, in proportion as the work of utero-gestation draws nearer to its close. Indeed, during its earlier stages, the traces of them are with difficulty recognizable; as the hæmorrhage, under these circumstances, is rarely profuse, and the uterus and abdomen but little distended. However distinctly visible they may have at first been, these signs commonly disappear in about ten days after the event with which they are connected has taken place.

We have before mentioned, that all these signs, taken isolately, may be fallacious, and that they only derive solidity from combination and perfect coincidence. Some objections to implicit reliance upon the results of anatomical examination have been already stated. It may be right to consider farther the identity of some of these signs with the phænomena of other and very different diseases.

In the first place, *the wrinkling and flaccidity of the abdominal integuments* may be the consequence of peritonæal dropsy, of long continued tympanites, of tumours seated in the hypogastric or umbilical regions, or of other cause totally unconnected with abortion.

Secondly. *Secretion of milk* may arise from mere suppression of the menstrual discharge; but the fluid, in this case, will be more watery, and the mammæ less pendulous and flaccid, than after abortion.

Thirdly. All the symptoms of pregnancy may result from an imperforate state of the hymen, and consequent retention and accumulation of the menstrual blood; and all the external phænomena, characterizing abortion, from rupture of the membrane and evacuation of the retained fluid.

Fourthly. Nothing can be more uncertain than the inferences drawn from the *condition of the uterine orifice.* In some young women, the size of this orifice is naturally as large as in women who have been recently delivered: or the alterations which it exhibits may depend on some organic lesion, or other cause very different from parturition. On the other hand, it must be allowed that, in persons who are pregnant for the first time and have suffered abortion at an *early* period of utero-gestation, the wrinkling and flaccidity of the skin of the abdomen does not take place;—that, in some females, especially those who have been nurses, the mammæ very tardily display the changes operated by gestation;* that the destruction of the fœtus *in utero* may be accomplished without the ordinary symptoms of abortion being developed, as when, the membranes having been penetrated, and yet the pla-

* Foderé has observed the mammæ of pregnant women "perfectly flaccid" between the fourth and fifth month. See his *Traité de Médecine Légale.* Tome iv.

centa not detached, the fœtus perishes, and its membranes, shrinking, separate gradually from the internal surface of the womb without producing hæmorrhage; and, lastly, that the uterine orifice sometimes preserves after delivery its previous regularity of figure, or even exhibits an additional degree of straitness and constriction.

Abortion, we shall observe in concluding this part of our subject, does not always immediately succeed the infliction of the violence by which it has been determined. Sometimes the causes by which the placenta is detached, do not suffice to expel the fœtus and secundines from the cavity of the uterus. Hæmorrhage necessarily ensues; but the constriction of the cervix uteri, while it allows the issue of the blood, prevents the escape of the solid and voluminous ovum. Thus the unadhering fœtus may be retained in the womb until the hæmorrhage has completely ceased, and that enfeebled organ re-acquired its power of contraction; and, although detached by violent means, be at last expelled without any attendant hæmorrhage. To discover the truth, under such circumstances, when there is reason to suspect provoked abortion, we must have recourse to the anterior signs, and to those which succeed the hæmorrhage. If it have been but a simple loss of blood, the health of the woman will be re-established in its cessation. The detached ovum, on the contrary, acting as a foreign body, will prove a constant source of irritation to the uterus, until expelled from its cavity. The subsidence of the abdomen, the softness and flaccidity of the mammæ, the fainting without apparent cause and transient shiverings, experienced by the woman, and discharge of black fœtid substances from the vagina, indicate sufficiently the presence of a detached and putrescent body within; and this indication will be eventually confirmed by the evacuation of the putrid mass from which the train of symptoms has originated.

We now proceed to examine the queries proposed in the commencement of this article, and to indicate the points, by attention to which their solution will be best undertaken and most effectually accomplished. In so doing, we shall deviate some little from our former arrangement.

First. *Has the fœtus been prematurely expelled, or, in other words, has abortion, or premature delivery, taken place.* To decide this question, it is absolutely requisite that the expelled substance be submitted to examination; for this constitutes the *corpus delicti;* in the absence of which all judicial proceedings fall to the ground; even although the woman suspected should avow her pregnancy, and evident traces of recent parturition be discovered upon her. This examination will prove more difficult and uncertain in proportion as pregnancy is less advanced. In fact, as Foderé very judiciously observes, juridical researches on abortion can scarcely be exercised before the close of the second month of utero-gestation, both because women, at first uncertain with respect to their situation, rarely attempt to procure abortion before this period; and till then it is very difficult to collect an assemblage of signs sufficiently well marked, in proof of its occurrence.*

We deem it needless to trace with all the minuteness and prolixity of description of the continental writers, the progressive developement of the various parts and organs of the human fœtus from the moment at which its slender rudiments first become visible to its entry upon a new and independent state of existence. It will be sufficient for our purpose to state that, observed between the fourth and sixth week from conception, the fœtus appears to be about the size of a wasp. The head forms then more than half its bulk; the eyes and mouth are well-marked; the hands and feet seem to be immediately attached to the trunk; the arms, legs, and thighs are scarcely visible. The containing ovum, about the seventh week, equals in volume a large hen's-egg. This ovum (independently of the double decidua by which it is invested as the head by a double night-cap) consists of two membranes; the external, named chorion, of a spongy structure and furnished outwardly with a very thick down; and the internal or amnios, thin, transparent, and displaying amid the limpid water which it contains, the body of the fœtus. These membranes adhere less intimately together at the commencement of pregnancy, than the chorion to the uterus. Hence, we sometimes see them disunited in early abortion and separately discharged. The chorion then frequently lodges on the orifice of the womb; and the amnios, inclosing the waters and fœtus, comes away entire; while some time elapses ere the former is expelled. In this case, the woman voids a kind of membranous ovum, which has not the least appearance of flocculi; and the chorion, when afterwards discharged, unless it be attentively examined, may be mistaken for a coagulum of blood, inasmuch as it is covered by a layer of this fluid.† From this period, the numerous and intimate connections of the chorion with the internal surface of the womb, the decided formation of the placenta; and the close relations of the fœtus with its membranes by means of the umbilical cord, render the traces of abortion, and the presence of the expelled ovum amid the accompanying coagula of blood, much less difficult of detection. In fact, the flocculi which have been described as projecting from the external surface of the chorion, soon become a thick, bloody mass, intimately attached to the uterus, and embracing the ovum in the greater part of its extent. If this mass be examined in a state of detachment from the uterus, we readily observe the bloody portion, more extensive in an inverse ratio to the term of pregnancy, embracing a vesicle that contains the fluid in which the embryo floats, not exactly at the centre, but towards one of the extremities of the mass.‡

---

* Foderé. Work before cited.

† Baudeloque. *L'art des Accouchemens.*

‡ Foderé. Work before quoted.

The following constitute the chief external signs by which the prematurely delivered may be distinguished from the mature fœtus.

*First*, The body of the former is emaciated, and dry; the skin, flaccid and loose, is nearly or altogether destitute of adipose substance: the blood, seen through the cutis, gives it a red or even purple colour, especially where it is more delicately organized, as in the palms of the hands and soles of the feet. *Secondly*, The small hairs which cover the skin of the mature fœtus, are scarcely visible: the sebaceous matter of the surface is less adherent, and resembles a flocculent down, which occupies particularly the lateral parts of the face, the back, loins, and shoulders. *Thirdly*, The fontanelles are generally very large and the cranial bones mobile; but to the first of these characters there exist some exceptions. *Fourthly*, The face is little developed, and exhibits an aged, unpleasing, sorrowful appearance; the lips and ears are purple; the tongue very red. *Fifthly*, The hair is thin, short, white or silvery; the nails scarcely apparent or totally wanting. *Sixthly*, The eye-lashes and eye-brows are scanty; the lids adhering together; the pupils covered by a membrane. *Lastly*, The scrotum is purple, much wrinkled, and commonly without the testes: the labia pudendi, in the female, are swollen; the nipples not larger than a pin's-head, and destitute of the wonted areola.*

The dimensions and weight of the fœtus are too variable to admit of precise specification. In general, the full-grown child weighs from seven to upwards of eight pounds; and measures, in length, from nineteen to twenty-two inches.

These data it will be right to bear in mind; but they are applicable only in combination with all the other signs. In proportion as utero-gestation is less advanced, and as a longer space of time has elapsed from the expulsion of the fœtus, the difficulties and uncertainty of the examination of the woman must necessarily be increased. On these occasions, it is very necessary to inspect the linen of the accused; and the appearance of blood and coagula thereon will fully justify prosecution of the research. The assertion, often brought forward by women, that such appearances have been caused by a copious return of the suppressed menstrual flux, although sometimes correct, should never be implicitly received by the juridical physician, or considered as rendering unnecessary a farther investigation.

Moles, or unorganized masses, inducing all the usual phænomena of pregnancy and parturition, are sometimes discharged from the uterus; but in these, the bloody portion, which the human ovum exhibits externally, is commonly found interiorly placed, and enveloped in a strong false membrane, the production of the albu-

* Marc. *Dictionnaire des Sciences Medicales.*

minous part of the blood. This may generally be regarded as a consequence of impregnation; and, when occurring in an unmarried female, might tarnish her character, or even implicate her in horrible suspicions of secret delivery and infanticide, although it would not endanger her life: for the English law requires, that "the body of the child be found before the coroner can hold an inquest." In such cases, a mark, resembling that of the placental attachment, would be seen on examination of the uterus. The very age of a person, in whom a flooding, which assumes the characters of abortion, may have taken place, will sometimes suffice to remove every rational suspicion of such an event.*

II. *Supposing the fact of abortion to be proved, has it been spontaneous, or voluntarily and criminally provoked?* In solving this momentous question, the confrontation of the moral circumstances with the physical effects of the action, can alone lead to a kind of certainty; and, although such research falls rather within the province of the jury than the physician, it, nevertheless, constitutes the only mean whereby he is enabled to dissipate the obscurity which envelopes the subject. The truth of this principle will be demonstrated by examination of the following points. They consist in determining whether the suspected female have suffered any fall, or other injury, followed by abdominal commotion; whether the abdomen present any bruises, or the fœtus any brown or livid spots; whether the injury were directly or shortly succeeded by hæmorrhage or other sign of disorder; whether the woman were of a temperament so irritable as to favour the operation of such violence; whether she have carried any burden, or made other effort injurious to the state of pregnancy, and at what period of this state, and what distance of time from the expulsion; whether there exist any internal cause capable of producing hæmorrhage, and whether it and the abortion have been preceded by a decidedly plethoric condition, or inordinate corporeal exercise; whether the woman have unduly compressed the abdomen with stays or ligatures, or, with a view of re-exciting the menstrual flux, have employed medicinal remedies; at what time the hæmorrhage began, its duration, and probable amount; and, lastly, whether the woman exhibit any irregularity or morbid condition?

The affirmative of a majority of these queries would exclude all idea of provoked abortion; which, on the contrary, would be sanctioned by concurrence of the following circumstances:—Has the woman, in question, concealed her pregnancy? Has she sought information on the various means of procuring abortion; or used, without necessity, violent and dangerous exercise? While in the enjoyment of good health, has she been busied with preparations and arrangements indicating an expectation of illness? Has she repeatedly, and in a private manner, requested different surgeons

---

* See Male and Foderé, *Works before quoted.*

to bleed her, particularly in the foot, without informing them of her having frequently been bled before; or, has she sought to obtain from any medical practitioner, or other person, articles proper for provoking hæmorrhage? Has she elsewhere procured this kind of drugs, or employed compounds of them prepared by herself? Has she, needlessly and without the prescription of a medical adviser, taken drastic purgatives; or have the remains of suspected drugs been found in her possession? Has she suddenly affected illness in order to conceal her state, and especially the uterine hæmorrhage subsequently discovered. Do there, lastly, exist on the generative organs of the mother, or body of the fœtus, marks indicating the direct infliction of violence by blows, or the puncture or laceration of sharp instruments, introduced by the vagina? All these, and other analogous circumstances, or greater part of them, being combined, announce premeditation, and invest with an undoubted criminal character, the fact of abortion. It ought, however, never to be forgotten that, certain direct operations excepted, most of the other remedies and circumstances may have been employed, or have taken place, without the most distant intention, on the part of the woman, to procure abortion. Of this we have witnessed several unquestionable instances.

III. *Was the fœtus, at the period of employment of the expulsive means, endued with life?* In resolving this last query, we shall refrain from all idle discussions respecting the period of animation of the fœtus; for the existence of organic life alone establishes vitality, and this dates from the moment of conception. It is here absolutely requisite that the body be examined: and, if this present depression of the fontanelles without traces of external violence, separation of the epidermis in many places, livid colour of the skin, a cadaverous smell, an almost pulpy softening of the muscles and viscera; a shrivelled, tender, fœtid, condition of the umbilical cord; a puffiness of the surface of the body, especially of the face and abdomen; we may conclude that the fœtus has perished anteriorly to its expulsion, without, however, being able to indicate precisely the period of death, or establish with certainty, in all cases, whether it have been determined by the employment of any peculiar means. Thus, for instance, if abortives have been used on the days just preceding abortion, and the mother have not felt the motions of her child for some time previously to such application, prudence and humanity would alike suggest the most favourable construction.—From what has been just said, it results, that the resolution of our third query is only practicable when the space of time, which has elapsed from expulsion to examination of the fœtus, is not sufficiently considerable to admit the supposition of extra-uterine putrefaction. The developement of this process is variable, and can only be appreciated, in an approximative manner, by a comparison of different circumstances.

We shall terminate this article in our ensuing number. EDIT.

